# Receptor Ligands as Helping Hands to L-DOPA in the Treatment of Parkinson’s Disease

**DOI:** 10.3390/biom9040142

**Published:** 2019-04-09

**Authors:** Fabio Del Bello, Mario Giannella, Gianfabio Giorgioni, Alessandro Piergentili, Wilma Quaglia

**Affiliations:** Scuola di Scienze del Farmaco e dei Prodotti della Salute, Università di Camerino, Via S. Agostino 1, 62032 Camerino, Italy; fabio.delbello@unicam.it (F.D.B.); mario.giannella@unicam.it (M.G.); alessandro.piergentili@unicam.it (A.P.); wilma.quaglia@unicam.it (W.Q.)

**Keywords:** Parkinson’s disease, levodopa therapy, levodopa-induced side effects, dopaminergic drugs, non-dopaminergic receptor ligands

## Abstract

Levodopa (LD) is the most effective drug in the treatment of Parkinson’s disease (PD). However, although it represents the “gold standard” of PD therapy, LD can cause side effects, including gastrointestinal and cardiovascular symptoms as well as transient elevated liver enzyme levels. Moreover, LD therapy leads to LD-induced dyskinesia (LID), a disabling motor complication that represents a major challenge for the clinical neurologist. Due to the many limitations associated with LD therapeutic use, other dopaminergic and non-dopaminergic drugs are being developed to optimize the treatment response. This review focuses on recent investigations about non-dopaminergic central nervous system (CNS) receptor ligands that have been identified to have therapeutic potential for the treatment of motor and non-motor symptoms of PD. In a different way, such agents may contribute to extending LD response and/or ameliorate LD-induced side effects.

## 1. Introduction

Parkinson’s disease (PD), also known as idiopathic paralysis agitans, is one of the most frequent chronic neurodegenerative diseases worldwide. Although its etiology has not been determined so far, the main pathological characteristic is the decrease of the dopamine (DA) level due to the degeneration of the dopaminergic neurons in the substantia nigra pars compacta [1,2]. This leads to motor (i.e., postural instability, dyskinesias, tremor, and rigidity) and non-motor (i.e., depression, cognitive impairment, pain, hallucinations) symptoms [3,4,5,6,7,8,9,10,11,12,13]. Another pathologically severe aspect is the abnormal formation of protein aggregates inside nerve cells (Lewy bodies), whose primary structural component is the presynaptic neuronal protein α-synuclein. For this reason, PD is classified as synucleopathy. Unfortunately, effective inhibition of progression or the cure for PD is not yet available, while all the available therapies only provide relief for symptoms.

Dopaminergic medications are currently the most effective treatment for both motor and non-motor symptoms, though they are not devoid of limitations and frequently produce undesired side effects. The standard treatment of PD patients consists in the administration of DA) in the form of levodopa (LD), a catecholamine produced by the intraneuronal tyrosine hydroxylation [14,15,16,17,18,19]. Its combination with a peripheral DOPA decarboxylase inhibitor (i.e., carbidopa) increases LD availability in the central nervous system (CNS) and ameliorates the therapeutic profile of LD, prolonging its efficacy [20,21,22]. An increase in the efficacy of dopaminergic therapy is also obtained by the simultaneous blockade of the DA metabolism with monoaminooxidase B (MAO-B) and/or catechol-O-methyl transferase (COMT) inhibitors [23,24]. Although LD represents the “gold standard” of PD therapy [25], unfortunately, orally administered LD can cause side effects, including gastrointestinal and cardiovascular symptoms as well as transient elevated liver enzyme levels. Moreover, LD therapy leads to LD-induced dyskinesia (LID) [26], a disabling motor complication that represents a major challenge for the clinical neurologist [27]. Indeed, LID negatively affects the quality of life [28,29,30] and constitutes a serious obstacle to the management of PD imposing a limit and a reduction of LD dosage, thus restricting treatment efficacy [27].

Numerous therapies are currently being developed to treat the motor and non-motor complications of PD and LID [31]. (See Appendix A for the PubChem CIDs (or Reaxys IDs) of the compounds reported in the review).

Mostly a customized combination of DA agonists and LD formulations is performed. The striatal D_1_ and D_2_ receptors are the common binding sites of DA ligands for PD treatment, but lately D_3_ and D_4_ subtypes have also become potential targets.

**Figure 1:**
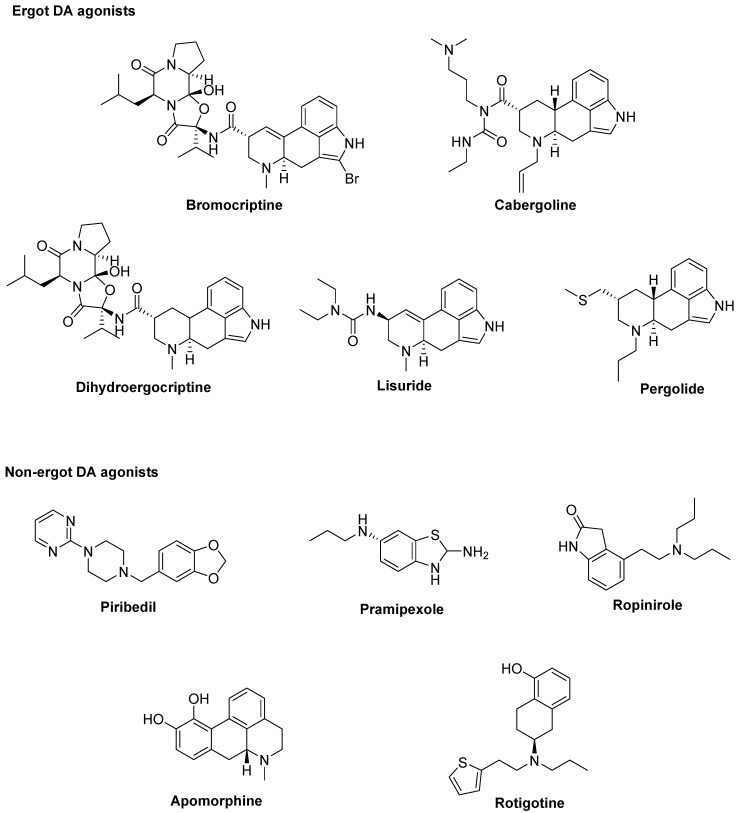
Dopamine (DA) agonists available for Parkinson’s disease (PD) treatment.

At the best of our knowledge, ten DA agonists are so far available for this disease. They can be listed in ergot DA agonists, including **Bromocriptine**, **Cabergoline**, **Dihydroergocriptine**, **Lisuride**, and **Pergolide** and non-ergot DA agonists, including **Piribedil**, **Pramipexole**, **Ropinirole**, **Apomorphine,** and **Rotigotine** [32] (Figure 1). 

**Figure 2:**
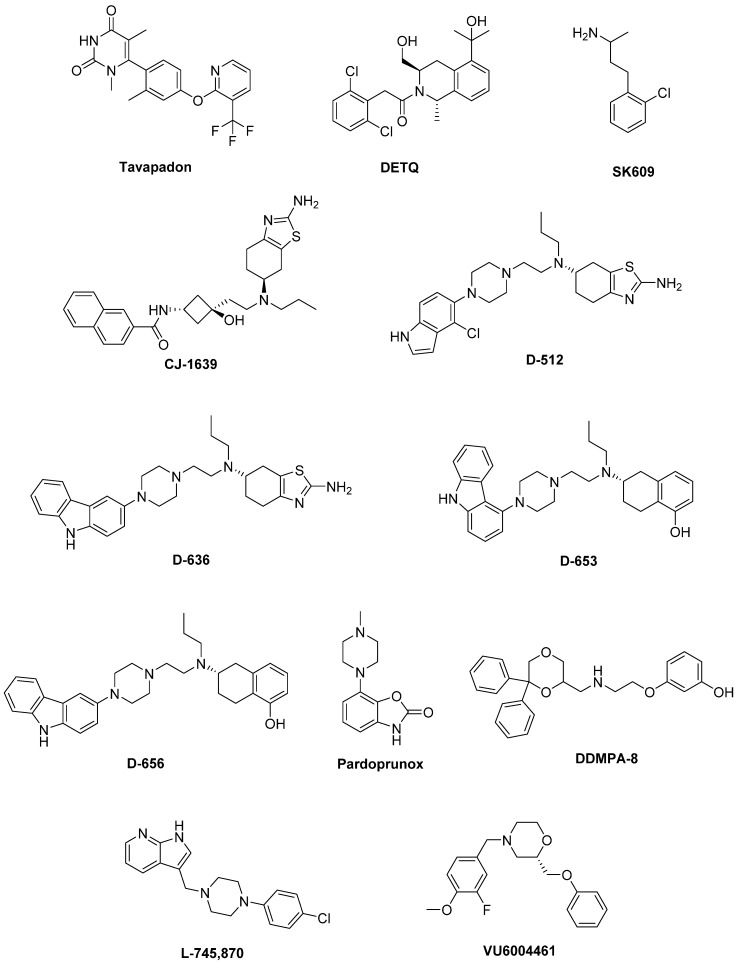
Emerging dopaminergic ligands as new levodopa (LD) adjuvant candidates.

Unluckily, DA agonists are not devoid of significant side effects such as hallucinations, hypotension, nausea, vomiting, pathological gambling, compulsive shopping and hypersexuality [33,34]. As a therapeutic example, symptoms of early stage PD may be controlled by the treatment with Pramipexole [35], but after a while a combination with LD is needed to optimize the management of PD symptoms [36]. Thus, DA agonists are typically used either to reduce the dosage of LD or to delay its use (LD sparing) [37], although it has been discussed that dyskinesia evolvement is due to disease persistence rather than protracted LD use [38]. Recently, research on dopaminergic targets has produced some new interesting candidates (Figure 2).

Among these, **Tavapadon** (or PF-06649751) is a novel, highly selective D_1_/D_5_ agonist. A recent paper, reporting about Phase I PD studies, candidates Tavapadon as a novel therapeutic agent for PD with an initial safety, tolerability, and pharmacokinetic profile as well as potential for efficacy. The same report asserts that Phase II clinical trials have been initiated to deeper investigate the potential safety and efficacy of Tavapadon with the aim to determine the dose that can produce relief of symptoms while reducing dependence on LD and, in the meanwhile, avoiding the problems associated with long-term LD administration [39].

Preclinical and clinical studies have indicated the potential utility of D_1_ agonists for the treatment of neuropsychiatric disorders. However, these agents are not devoid of limitations. For instance, it has been demonstrated that LID results from increased D_1_ receptor-mediated transmission at the level of the direct pathway. Moreover, unlike positive allosteric modulators (PAMs), orthosteric D_1_ receptor agonists produce receptor desensitization and an inverted U-shaped dose-response curve [40]. The development of the D_1_ PAM **DETQ** has been reported as a different approach to D_1_ receptor activation [41]. Being able to amplify the effects of released endogenous DA in situ, DETQ gives a more physiological response. Its CNS pharmacology strictly reminds that of D_1_ agonists, but also shows remarkable differences (i.e., it does not induce stereotypy or desensitization) [41]. The reported behavioral and neurochemical test results suggest a therapeutic utility in neuropsychiatric disorders such as PD [42,43]. 

It has also been hypothesized that DA receptors in the striatum can form heteromeric complexes. Such an heteromerization leads to changes in the functional and pharmacological properties of receptors compared to their monomeric subtypes [44,45]. It has been observed a correlation between the expression of D_1_–D_3_ receptor heteromers and the development of LID [46]. Furthermore, D_3_ receptor stimulation can potentiate the D_1_ receptor signaling pathway [46,47]. Thus, future D_3_ antagonists or partial agonists able to selectively modulate the activity of striatal D_1_–D_3_ receptor heteromers could be very promising in LID control [48]. Treatment with LD also induces an ectopic expression of D_3_ receptors in the DA depleted dorsal striatum, which is associated with dyskinesia [40,46,49]. D_3_ receptor involvement in dyskinesia has further been proved by PET studies in humans showing an elevated D_3_ receptor binding in patients with dyskinesia [50]. It has been reported that D_3_ receptor agonists may produce neuroprotective effects by directly scavenging free radicals, improving the activity of free radical scavenging enzymes, stabilizing the mitochondrial membrane, directly inhibiting neuronal apoptosis. Moreover, being D_3_ receptors primarily localized in the midbrain limbic system, which is unrelated to motor function, selective D_3_ receptor agonists may have suitable anti-PD activity without significant extrapyramidal side effects [51,52,53,54,55].

The D_3_ receptor subtype has also been shown to exhibit biased signaling and desensitization pattern in response to certain agonists, DA included. Such an evidence could significantly contribute to the development of motor and hyperkinetic symptoms in PD and LID, respectively. On the contrary, the closely related D_2_ receptors have not demonstrated these D_3_ characteristics [40,56]. Thus, it has been demonstrated that the selective D_3_ agonist **SK609**, which does not induce desensitization of D_3_ receptors in vivo [57,58], was able to decrease locomotor activity [59,60]. Moreover, it has also been observed a dose dependent efficacy of SK609 in improving motor deficits in PD and ameliorating abnormal involuntary movements (AIMs) in LID using the hemiparkinsonian unilateral lesioned rodent PD model. A combination of SK609 and a low dose of LD induced a motor symptomatic relief without producing AIMs [61]. 

**CJ-1639** is actually one of the most potent and selective D_3_ full agonist reported to date that may become one of the newer anti-PD drugs [62,63].

The novel ‘multifunctional’ D_2_/D_3_ high-affinity compound **D-512**, endowed with receptor agonist activity together with antioxidant and other neuroprotective features has recently been developed [64,65]. Compared with Ropinirole, it showed greater peak-dose efficacy and a longer lasting action, thus deserving consideration for clinical investigation. 

The novel carbazole-based multifunctional D_2_/D_3_ receptor ligands **D-636**, **D-653,** and **D-656**, endowed with high binding affinity and full agonist activity at both receptors [66], have been proved to be highly efficacious in a PD rat model indicating their potential in relieving motor dysfunction in PD. They also exhibited neuroprotective property in an in vitro cellular model of PD. Furthermore, D-636 and D-653 demonstrated potent modulator effect on aggregation and toxicity of α-synuclein protein in vitro. Thus, it has been postulated that multifunctional drugs like D-636, D-653, and D-656 have the potential to alleviate motor dysfunction in PD patients, as well as to modify the disease progression.

**Pardoprunox** (SLV-308), a D_2_/D_3_ receptor partial agonist and 5-HT_1A_ receptor full agonist, reached Phase III clinical trials for the treatment of PD. Compared with other dopaminergic agents, it displayed lower propensity to elicit side effects like dyskinesia [67].

Since ligands endowed with such a multitarget profile might be effective in PD pharmacotherapy, novel multitarget compounds based on the *N*-((6,6-diphenyl-1,4-dioxan-2-yl)methyl)-2-phenoxyethan-1-amine (DDMPA) scaffold were studied. Interestingly, the 3-hydroxy derivative, here named for the first time **DDMPA-8**, behaved as a partial agonist at D_2_ and as a potent full agonist at D_3_ and D_4_ subtypes. In addition to its potent 5-HT_1A_ receptor agonism, that might be helpful in reducing dyskinetic side effects associated with the dopaminergic stimulation, such a dopaminergic profile makes DDMPA-8 a potential multitarget compound for the treatment of PD. In perspective, its evaluation in PD animal models would shed light on its therapeutic potential [68].

D_4_ receptors are present within the basal ganglia that represent a key area involved in parkinsonism and, in particular, in dyskinesia [69,70]. During the last years, a renewed interest has emerged around D_4_ receptors as potential therapeutic target for the treatment of PD, in which D_4_ antagonists can attenuate LID [71,72,73,74]. It has been observed that associating the potent D_4_ antagonist **L-745,870** to LD significantly ameliorates the dyskinesia scenario in LID models. Such a result was quite remarkable since this compound has also demonstrated to be well tolerated in clinical trials. Thus, it could have a rapid development as a new tool for LID treatment. Unfortunately, disappointing results were obtained in the rotarod performance test when co-administered with LD. In fact, L-745,870 reduced the overall LD antiparkinsonian benefit in this model opening only a narrow therapeutic window to its use for the treatment of LIDs [75,76].

The effect of the novel selective D_4_ antagonist, **VU6004461** [77], endowed with high blood–brain barrier penetrability has also been investigated. The clear antidyskinetic effect of both L-745,870 and VU6004461 points to the D_4_ as a possible future target for the treatment of LID [78]. At present more work is needed, but the use of D_4_ antagonists for the treatment of LIDs in PD remains a very promising area of research and the development of more highly optimized ligands is still an acceptable challenge [73]. 

All the results obtained so far are not enough and the rising of the aged population imposes new strategies in PD that may help to manage known limitations of current therapies. Some of the alternative strategies investigated as potential treatment of LID in PD involve non-dopaminergic receptors. To help researchers in such a challenge, this review focuses on recent investigations about non-dopaminergic CNS receptor ligands that have been identified to have therapeutic potential for the treatment of motor and non-motor symptoms of PD. Such agents in different way may contribute to extend LD response and/or ameliorate LD-induced side effects.

## 2. Serotonin Receptors

The serotonin (5-HT) system has been demonstrated to play a crucial role in the pathogenesis of LID in animal models of PD [79]. Indeed, after advanced dopaminergic cell loss, remaining serotonin neurons can convert exogenous LD to DA and mediate its vesicular storage and release [38,80]. The non-physiological DA release from these neurons might cause DA receptor overstimulation, leading to generation of dyskinesia [81]. Consequently, modulation of 5-HT system has emerged as a promising strategy for LID management. Several studies have shown a reduction of LID induced by targeting different 5-HT receptor (5-HTR) subtypes. 5-HT_1A_R (dorsal raphe nucleus and striatum), 5-HT_1B_R (striatopallidal pathways), and 5-HT_2A_R (substantia nigra pars reticulata and internal segment of the globus pallidus) can modulate DA, GABA, and glutamate release within the basal ganglia to improve motor symptoms of PD and to reduce dyskinesia [82]. 5-HT_1A_R and 5-HT_1B_R agonists, as well as 5-HT_2A_R and 5-HT_3_R antagonists have demonstrated a potential as antidyskinetic agents, while 5-HT_4_ agonists can increase LD-stimulated DA release in CNS.

### 2.1. 5-HT_1A_Rs

5-HT_1A_R is the most studied of the 5-HT family. Indeed, several preclinical and clinical studies demonstrated that 5-HT_1A_R stimulation (auto- and heteroreceptors) [49,83,84] may reduce dyskinesia through the decrease of DA release [79]. Moreover, 5-HT_1A_R activation may also weaken glutamatergic transmission ameliorating motor symptoms [83]. 

**Figure 3:**
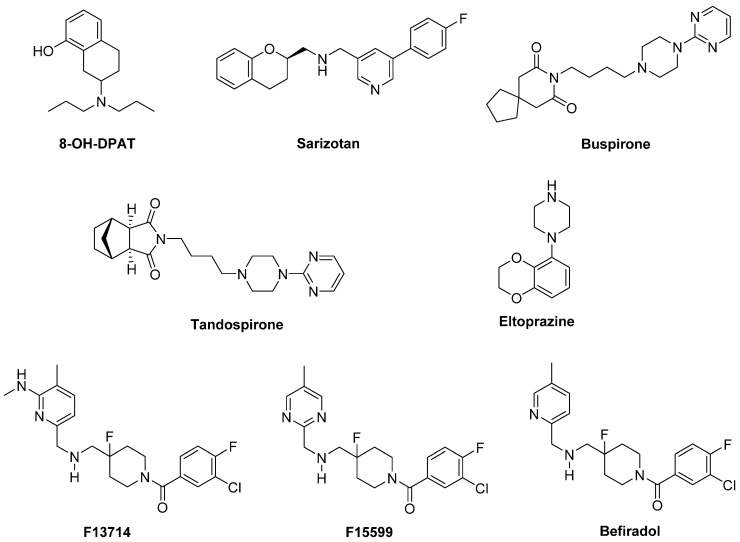
5-HT_1A_R agonists.

In a preclinical study, the highly selective 5-HT_1A_ full agonist **8-OH-DPAT** and its (*R*)-(+) eutomer reduced LID, but also worsened motor function in a 1-methyl-4-phenyl-1,2,3,6-tetrahydropyridine (MPTP)-lesioned primate model [85] (Figure 3). 

**Sarizotan**, another 5-HT_1A_R full agonist, also endowed with partial D_2_-like agonist/antagonist profile [86], showed better results as an antidyskinetic agent in a 6-hydroxydopamine (6-OHDA)-lesioned rat model of PD [87]. When evaluated in a Phase II clinical study, at low dose it ameliorated some PD symptoms [88]. However, it failed in attenuating LID compared with placebo in two Phase III studies [89], limiting its therapeutic potential.

The partial 5-HT_1A_R agonists **Buspirone** and **Tandospirone** have been shown to possess antidyskinetic properties in humans, but also negatively impacted on parkinsonian symptoms [90,91]. Currently, a Phase III clinical trial is investigating the antidyskinetic potential of Buspirone, which also behaves as a D_2_-like receptor antagonist [92], on LID (NCT02617017), while a Phase I clinical trial is exploring its potential in combination with the non-selective NMDA antagonist Amantadine (NCT02589340).

Another 5-HT_1A_R partial agonist able to reduce LID in combination with LD in preclinical studies is **Eltoprazine** [93]. Unlike Buspirone and Tandospirone, this compound also behaves as a 5-HT_1B_R agonist and a 5-HT_2C_R antagonist [94]. In both rodent and monkey models, it abolished LD-mediated motor improvements, suggesting that it may have a narrow therapeutic window [93]. However, the loss of LD efficacy proved to be mitigated by co-administration with 5-hydroxy-tryptophan [95]. When tested in a clinical Phase I/IIa study, Eltoprazine attenuated LID without affecting the antiparkinsonian action of LD [96]. To further validate its efficacy, another Phase II trial is currently ongoing to assess the duration of Eltoprazine’s efficacy in LID management and its effects on motor function (NCT02439125). Preclinical studies have also highlighted the potential efficacy of combining eltoprazine with other compounds able to attenuate LID, such as Amantadine and the selective adenosine A_2A_ receptor antagonist Preladenant [93,97,98]. 

The 5-HT_1A_ agonists so far evaluated in clinical trials have shown off-target effects and only partial agonist efficacy at 5-HT_1A_R. In this contest, the new highly selective 5-HT_1A_R biased agonists **F13714**, **F15599**, and **Befiradol** (also known as F13640 or NLX112) were recently demonstrated to exhibit exceptionally potent antidyskinetic activity in animal models of PD, while minimally interfering with LD antiparkinsonian effects [99,100,101]. Biased 5-HT_1A_ agonists are selective ligands that act in specific brain regions and preferentially target different 5-HT_1A_R subpopulations [102]. While F13714 and Befiradol preferentially bind presynaptic 5-HT_1A_ autoreceptors, F15599 activates postsynaptic 5-HT_1A_Rs [100,103,104]. Befiradol has recently been shown to possess a distinctive in vitro G-protein activation profile in rat brain cell membranes which differs from those of F13714 or F15599 [105]. In particular, it preferentially activate G_αo_ proteins over other G-protein subtypes. This compound is currently undergoing clinical development as an antidyskinetic agent (www.parkinsons.org.uk/news/investing-new-treatment-dyskinesia).

### 2.2. 5-HT_1B_Rs

**Figure 4:**
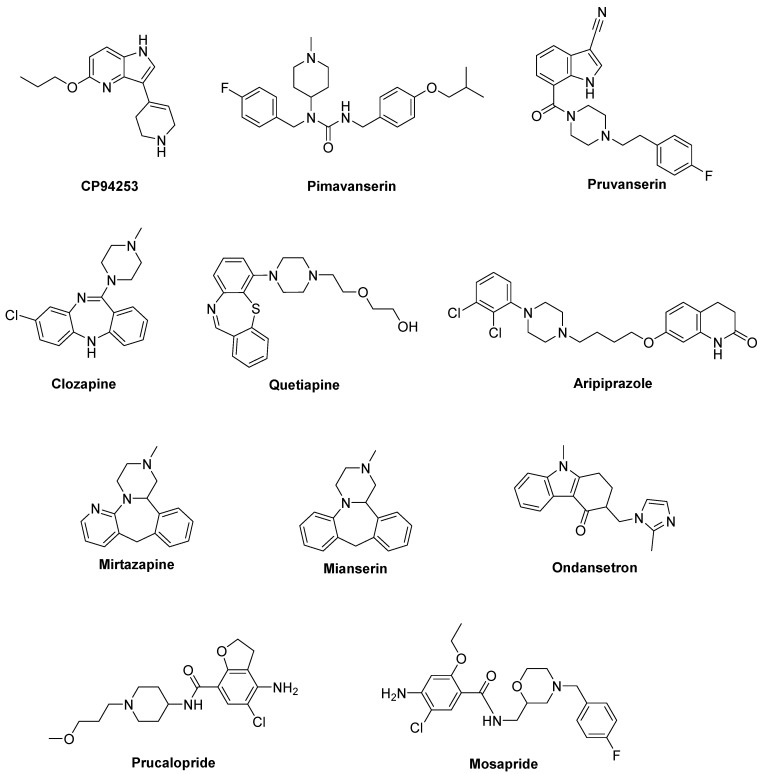
5-HT_1B_R, 5-HT_2A_R, 5-HT_3_R, and 5-HT_4_R ligands.

Although studies with compounds selectively targeting 5-HT_1B_R are limited, the selective 5-HT_1B_R agonist **CP94253** demonstrated to attenuate LID in a 6-OHDA-lesioned rat model [81,106] (Figure 4). Interestingly, CP94253 also reduced dyskinesia induced by D_1_ receptor agonists at low doses [106]. However, a definitive role of 5-HT_1B_R in LID is difficult to be defined due to very limited clinical studies. Instead, the combination of 5-HT_1B_R and 5-HT_1A_R agonism is more often studied both by monotherapies with mixed actions (e.g., the 5-HT_1A_R/5-HT_1B_R agonist Eltoprazine) or by combined therapies [81]. More recently, the co-administration of the 5-HT_1B_ agonist CP94253 with the 5-HT_1A_ agonist 8-OH-DPAT and the metabotropic glutamate 5 receptor (mGlu5R) antagonist MTEP elicited a great synergistic antidyskinetic effect without impairment of the antiparkinsonian effects [107]. 

### 2.3. 5-HT_2A_Rs

Among 5-HT_2_R subtypes, a potential role in PD and LID has been suggested for 5-HT_2A_R. **Pimavanserin** (ACP-103), a potent 5-HT_2A_R and less potent 5-HT_2C_R inverse agonist [108], demonstrated to attenuate the expression of LID in cynomolgus monkeys without reducing LD efficacy [109] (Figure 4). This compound has been approved in the United States for the treatment of dopamimetic-induced psychosis in PD patients [110]. However, clinical studies examining this compound against LID have not been reported so far. Evidence in support of Pimavanserin for the management of psychosis in PD patients comes from a Phase III placebo-controlled trial, showing that it was well tolerated and didn’t worse motor function [111].

The highly selective 5-HT_2A_ receptor antagonist **Pruvanserin** (EMD-281,014, LY-2,442,347) demonstrated to reduce the severity of LID and psychosis in a primate PD model, without affecting LD anti-parkinsonian activity [112]. On the contrary, it failed to reduce LD-induced AIMs in 6-OHDA-lesioned rat model, highlighting differences between rodent and primate models of PD [113].

Other evidences of 5-HT_2A_R involvement in LID derive from studies with antipsychotic compounds that are not selective towards such a subtype. For example, in both 6-OHDA-lesioned rat and MPTP-lesioned marmoset models, the atypical antipsychotic **Clozapine** reduced LID psychosis-like behaviors [114,115]. When tested in humans, Clozapine successfully reduced both duration and severity of LID symptoms without affecting LD efficacy [116,117]. However, the observation that Clozapine also displays affinity for other receptors, including 5-HT_2C_, D_2_ and D_4_, makes it difficult to evaluate the direct involvement of 5-HT_2A_R.

**Quetiapine**, another atypical antipsychotic agent targeting 5-HT_2A_R in addition to other systems including adrenergic, muscarinic, histaminergic, and dopaminergic receptors [118,119], effectively reduced LID without interfering with LD efficacy in 6-OHDA-lesioned rat and MPTP-lesioned macaque models [120]. Nevertheless, the results of clinical trials with this compound seem to be conflicting. Indeed, while a clinical study reported that low doses of Quetiapine did not significantly attenuate LID [121], another study found that it reduced LID with worsening of few motor symptoms [117].

The antipsychotic **Aripiprazole** is endowed with a multitarget profile, showing antagonism at 5-HT_2A_R and partial agonism at both 5-HT_1A_R and D_2_ [122]. In clinical studies it was able to attenuate hallucinations associated with PD, but also reduced LD efficacy in some patients [123]. Moreover, at a very low dose, it provided long-term LID relief [124]. 

Finally, both **Mirtazapine** and its analogue **Mianserin**, antagonists at noradrenergic receptors and 5-HT_2_R/5-HT_3_R [125], demonstrated to reduce LID in NHP models [126,127]. However, Mianserin also reduced LD efficacy, limiting its clinical use. In clinical trials mirtazapine was reported to reduce LID without worsening PD symptoms, particularly in patients that were non-responsive to Amantadine [128]. Further clinical studies are in progress. 

### 2.4. 5-HT_3_Rs

Recently it has been proposed that stimulation of the receptor channel 5-HT_3_R might affect DA release in striatum. The 5-HT_3_ antagonist **Ondansetron** decreased AIMs scores in 6-OHDA-lesioned rat model of PD, suggesting its efficacy in LID. However, it had no effects on motor coordination in rotarod behavioral test [129] (Figure 4).

### 2.5. 5-HT_4_Rs

In a recent study, the 5-HT_4_ agonist **Prucalopride**, evaluated in a 6-OHDA-lesioned rat model of PD, selectively enhanced LD-stimulated DA release in the substantia nigra pars reticulata and prefrontal cortex (Figure 4). The enterokinetic properties of 5-HT_4_R agonists suggested their potential use against LD-induced fluctuations in patients with PD [130]. Moreover, since 5-HT_4_ agonists displayed anxiolytic/antidepressant properties in a mouse corticosterone model [131], Prucalopride may represent an alternative approach to the treatment of anxiety and/or depression in LD-treated patients with PD [132]. 

The mixed 5-HT_3_R antagonist/5-HT_4_R agonist **Mosapride** proved to be effective in promoting the lower gastrointestinal tract motility and in ameliorating constipation in PD patients [133]. 

## 3. Glutamate Receptors

In rodent models of LID, high extracellular levels of glutamate were observed in the striatum and substantia nigra pars reticulata. Molecular imaging studies suggested that similar neurochemical changes of this system are evident in PD patients [134]. Therefore, glutamate receptors represent attractive targets for the treatment of LID. While the first efforts were addressed to antagonize the ionotropic glutamate receptors (iGluRs) N-methyl-D-aspartate (NMDA) and α-amino-3-hydroxy-5-methyl-4-isoxazolepropionic acid (AMPA) subtypes [135], more recently, metabotropic glutamate receptors (mGluRs) have also been considered as potential targets for PD and LID treatment [136,137].

### 3.1. iGluRs

Several studies performed in animal models of LID and in post-mortem basal ganglia tissues from dyskinetic PD patients have revealed modifications in the expression and state of phosphorylation of iGluRs, in particular NMDA and AMPA receptors [134]. Therefore, these receptor systems are considered of major importance to the pathophysiology of LID.

#### 3.1.1. NMDA Receptors

Alterations in NMDA receptor trafficking and distribution in the postsynaptic neurons appear to be associated with the extent of DA denervation as well as with the development of LID. However, the exact mechanisms regulating NMDA receptor subcellular trafficking and function in PD and LID are not fully elucidated yet [138,139]. Among the subunits forming the NMDA receptor, GluN2B subunit has attracted considerable interest. Indeed, from radioligand binding studies, performed both in NHP models of LID and dyskinetic PD patients, increased binding densities at GluN2B-containing NMDA receptors in the putamen were observed [140,141]. Furthermore, increased levels of GluN2B phosphorylation have been found in 6-OHDA-lesioned rats after chronic LD treatment [142].

**Figure 5:**
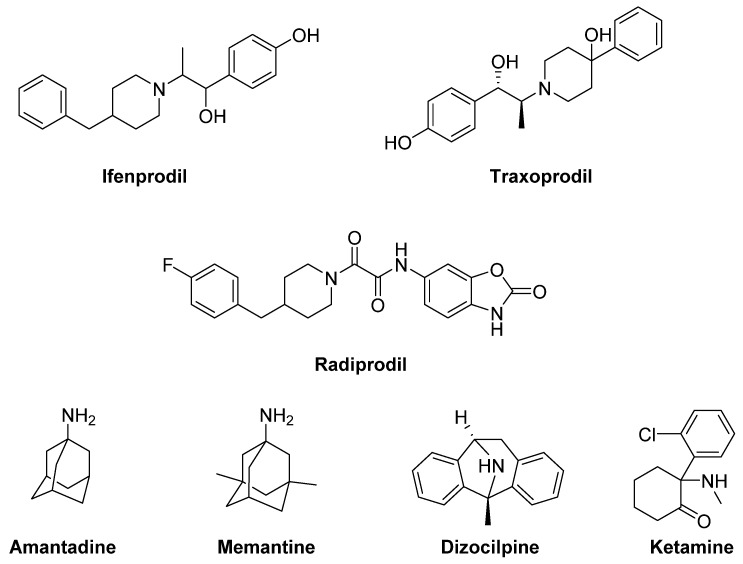
N-methyl-D-aspartate (NMDA) receptor ligands.

The GluN2B-selective antagonists **Ifenprodil** and **Traxoprodil** (CP-101606) were reported to ameliorate parkinsonian symptoms and to reduce LID in rat and NHP models [143,144,145,146,147,148] (Figure 5). However, their use has been discouraged in NHP owing to the development of severe side effects, including amnesia and dissociation [143,145,149,150].

In general, divergent results have been obtained following treatment with GluN2B-selective antagonists in animal models of LID, ranging from improvement to no effect, and even to a worsening of AIMs [134]. 

**Radiprodil**, another GluN2B-selective antagonist, in combination with the selective A_2A_ receptor antagonist Tozadenant, significantly improved motor activity both in 6-OHDA-lesioned rats and MPTP-lesioned NHP models, suggesting that the use of such a combination could lead to motor improvement to PD patients, without inducing the motor complications induced by LD therapy [151,152].

Promising results for the treatment of LID were also obtained with the weak non-competitive NMDA receptor antagonists **Amantadine** and **Memantine**. Amantadine, historically used as an antiviral agent, showed moderate but significant antidyskinetic efficacy in various clinical trials performed in the last two decades [153,154,155]. For this reason, it is the only drug with established antidyskinetic activity available in the market [156]. Amantadine treatment proved to reduce the duration of LID and to improve motor disability in PD [157] without major complications [154]. However, there are contrasting results concerning its long-term efficacy [158,159]. A Phase II clinical trial is currently ongoing to study the impact of Amantadine in preventing LID in early PD (NCT01538329). Other Phase II clinical trials are currently underway to evaluate the efficacy of Amantadine, in combination with other classes of drugs (e.g., Buspirone or Eltoprazine, see the section “Serotonin receptors”), in reducing LID in preclinical or clinical trials. Moreover, a recent study has revealed that the combination of a sub-effective dose of Amantadine and the nitric oxide synthase inhibitor 7-Nitroindazole potentiated the effect of reducing LD-induced AIMs in 6-OHDA-lesioned rats when compared to the effect of the drugs alone. This strategy may provide therapeutic benefits to PD patients at lower and thus more tolerable doses [160]. Memantine has also been investigated for its antidyskinetic potential in PD patients, but the results were conflicting. Although Memantine treatment was associated with lower LID scores and reduced daytime duration of dyskinesia, no significant effects on dyskinesia severity were found [161,162,163].

Other non-competitive NMDA receptor antagonists, including **Neu-120** (structure not disclosed), **Dizocilpine** (MK-810) and **Ketamine**, displayed potential antidyskinetic effects. 

Neu-120 is produced by Neurim Pharmaceuticals for the treatment of drug-induced dyskinesias. This compound, that also inhibits MAO-B and GSK-3β, has been subjected to a Phase I/II clinical trial to determine its safety, tolerability, pharmacokinetic and pharmacodynamic profiles in reducing LID in patients with advanced-phase idiopathic PD (NCT00607451). The study has been completed, but the results are not available yet. 

Dizocilpine also reduced LD-induced AIMs in a rat model of LID, but only at concentrations that worsen parkinsonism [164]. However, when this compound was co-administered with the opioid glycopeptide **Lactomorphin** (see Figure 17), its pro-parkinsonian activity was suppressed, while a strong antidyskinetic effect remained [165].

Finally, the dissociative anesthetic Ketamine, administered at low sub-anesthetic doses, displayed a long-term effect in reducing LID in a preclinical 6-OHDA-lesioned rat model [166]. This result was confirmed by a clinical trial, in which intravenous infusion of low doses of Ketamine induced a long-lasting therapeutic benefit to reduce LID and depression in PD patients [167].

#### 3.1.2. AMPA Receptors 

Analogously to NMDA receptors, synaptic localization and phosphorylation of AMPA receptors proved to be altered in animal models of LID and in PD patients [168,169,170,171]. Moreover, in MPTP-lesioned monkeys and 6-OHDA-lesioned rats, the pharmacological blockade of AMPA receptors decreased LIDs and enhanced the antiparkinsonian effect of LD [172,173,174]. Conversely, AMPA receptor agonists triggered dyskinesias [174]. In the light of these findings, treatments with selective AMPA receptor antagonists alone or in combination with selective NMDA receptor antagonists showed beneficial effect in reducing dyskinesia [172].

**Figure 6:**
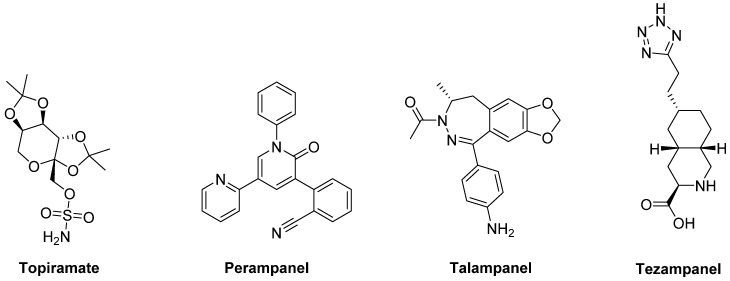
amino-3-hydroxy-5-methyl-4-isoxazolepropionic acid (AMPA) receptor ligands.

The anticonvulsants **Topiramate** and **Perampanel** are the only AMPA receptor antagonists which have reached clinical trials (Figure 6). Topiramate is a negative modulator of AMPA receptors [175] and a PAM of GABA_A_ receptors [176]. It has been reported to improve LID in MPTP-lesioned NHPs [177]. Moreover, in combination with the non-competitive NMDA receptor antagonist Amantadine, Topiramate elicited a synergistic antidyskinetic effect in both rodent and marmoset models of LID at low doses [178]. Despite these positive preclinical experiences, clinical trials have provided conflicting results. In a double-blind trial involving patients with idiopathic PD, Topiramate worsened dyskinesia and was poorly tolerated [179]. No results are so far available for other two Phase II clinical trials evaluating the efficacy of the combination of Amantadine and Topiramate versus Amantadine alone in PD patients with or without dyskinesia (NCT00794313, NCT01789047). Conflicting results were also found in clinical trials with the non-competitive antagonist **Perampanel** [180,181,182]. 

In preclinical studies, **Talampanel** (LY-300164, GYKI 537773), another non-competitive AMPA receptor antagonist, increased the anti-parkinsonian benefits of LD in MPTP-treated monkeys [174], while the competitive AMPA antagonist **Tezampanel** (LY-293558) was able to reduce wearing-off of LD-induced motor responses in 6-OHDA-lesioned rats [183].

### 3.2. mGluRs

mGluRs modulate intracellular signaling pathways without blocking the main action of glutamate on excitatory synaptic transmission. For this reason, they may be considered drug targets more convenient than iGluRs. Evidence demonstrated that mGluRs regulate pathophysiologically crucial events related to PD and LID [184,185].

#### 3.2.1. mGlu2/3Rs

**Figure 7:**
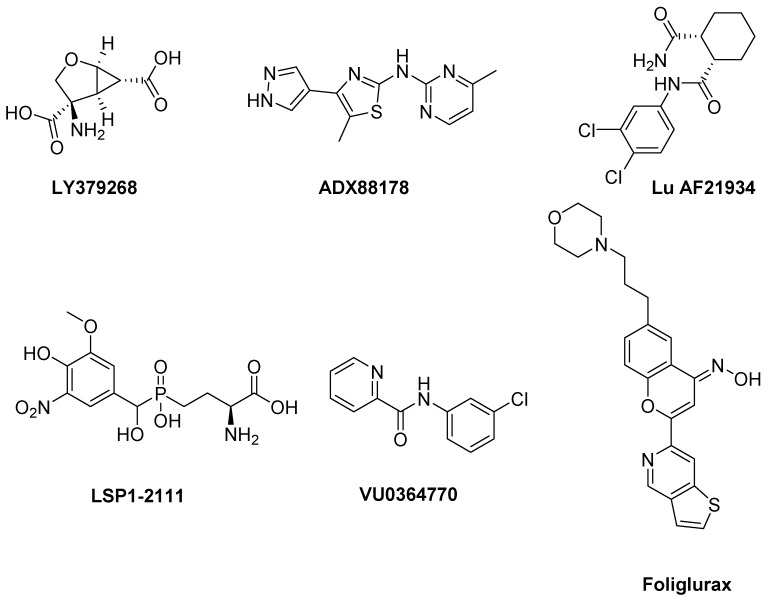
mGlu2/3R and mGlu4R ligands.

mGlu2R and mGlu3R agonists have been proposed to be used in the treatment of PD and LID [134]. In a reserpine-treated model of PD, the mGlu2/3R agonist **LY379268** was able to reduce akinesia [186] (Figure 7). However, this result was in contrast with that obtained in the 6-OHDA-lesioned model of PD, in which LY379268 failed to modify the bias towards ipsiversive rotations [186]. It should be considered that discrepancies between results from different PD models may depend on different degrees of DA depletion. LY379268 also failed to produce any amelioration of AIM scores in a rat model of LID [149]. Because of these contrasting effects, the use of mGlu2R and mGlu3R agonists for the treatment of PD and LID may be challenging.

#### 3.2.2. mGlu4Rs

Activation of mGlu4R using PAMs or orthosteric agonists induces antiparkinsonian effects in animal models of PD [187]. The mGlu4R PAMs **ADX88178** [188] and **Lu AF21934** [189] potentiated the effect of LD without increasing LID in a 6-OHDA-lesioned rat model (Figure 7). The above mentioned PAMs and the orthosteric agonist **LSP1-2111** [190], when co-administered with LD, showed LD sparing effect. This makes mGlu4R agonists potentially useful to allow LD to maintain the same benefit on PD motor symptoms al lower doses. Such an effect would indirectly improve LID. In a more recent study the observation that the stimulation of mGlu4R with the orthosteric agonist LSP1-2111 lacked antidyskinetic and LD-sparing activities while the PAM **VU0364770** decreased LID in 6-OHDA-lesioned rats demonstrated that an mGlu4R PAM might be an antidyskinetic agent better than an orthosteric agonist [191].

Recently, novel potent and selective mGlu4R PAMs with improved pharmacokinetic profiles after oral administration have been discovered. Among them, **Foliglurax** (PXT002331) fully reversed hypokinetic deficits in 6-OHDA-lesioned rats when co-administered with sub-threshold doses of LD [192] and proved to alleviate the motor symptoms of PD and the motor complications induced by LD in primates [193]. Foliglurax is currently being evaluated in Phase IIa trial (NCT03162874) in PD patients affected by LID and wearing-off fluctuations. These results support mGlu4R as a novel and promising therapeutic target for PD and LID.

#### 3.2.3. mGlu5Rs

Several preclinical and clinical studies demonstrated the involvement of mGlu5R in PD and LID. In particular, negative allosteric modulators (NAMs) have proven high efficacy to reverse motor deficits and inhibit LID in both 6-OHDA-lesioned rat and MPTP-lesioned NHP models of PD [184,185].

**Figure 8:**
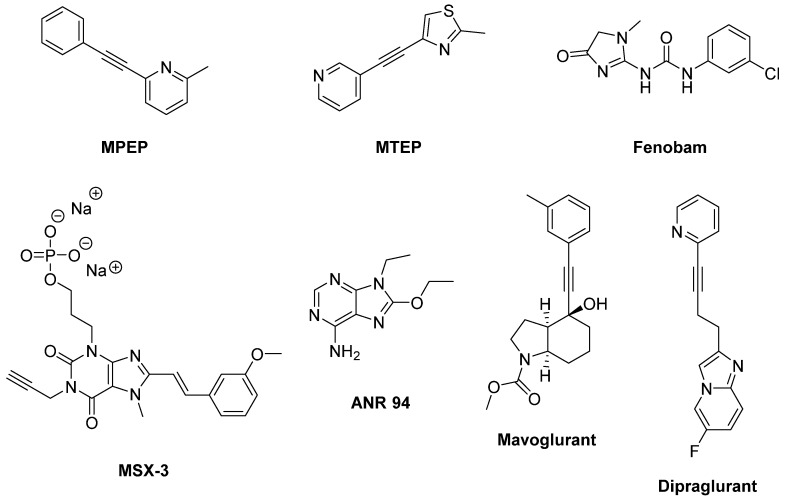
mGlu5R ligands.

The NAMs **MPEP** and **MTEP** attenuated the effects of LD in inducing involuntary movements in the 6-OHDA-lesioned rat model of LID (Figure 8) [194,195]. Accordingly, MTEP potently inhibited AIMs triggered by the D_1_ receptor agonist SKF38393 [107]. MPEP and MTEP also reduced the intensity of LID after acute administration in NHP models of PD [196]. Moreover, MPEP proved to be efficacious after chronic administration without affecting LD efficacy [197]. These results were in line with a previous study, in which the mGlu5R NAM **Fenobam** reduced LID in both 6-OHDA-lesioned rats and MPTP-lesioned monkeys [198]. Moreover, a combination of Fenobam and Amantadine at sub-threshold doses reduced LID without worsening PD [199], while a combination of MPEP and the adenosine A_2A_ antagonists **MSX-3** and **ANR 94** synergistically increased LD-induced turning [200].

Accordingly, in Phase II clinical studies (NCT00582673, NCT00888004, and NCT00986414) the mGlu5R NAM **Mavoglurant** (AFQ056) demonstrated antidyskinetic efficacy without worsening PD motor symptoms [137,201,202]. The most common adverse events were reported to be dizziness, hallucinations, diarrhea, and insomnia. Unfortunately, Mavoglurant failed to replicate the previous outcome in two subsequent Phase II clinical studies (NCT01491529, NCT01385592), leading to discontinue clinical trials of this compound for the treatment of LID [203]. In another clinical study (NCT01092065), administration of Mavoglurant in patients treated with high doses of LD avoided a worsening of dyskinesia. However, this study was limited by the reduced number of patients, the short treatment duration and the conflicting clinician-rated measures [204].

Among the mGlu5R NAMs, **Dipraglurant** (ADX48621) showed the most encouraging clinical results. Indeed, in a recent Phase II clinical trial it effectively reduced LID severity including a reduction of dystonia severity and chorea (two major LID components) with no evidence of worsening parkinsonism. Moreover, Dipraglurant demonstrated good safety and tolerability [205], deserving to be further investigated in a larger number of patients to confirm its efficacy in the treatment of LID.

#### 3.2.4. mGlu7Rs and mGlu8Rs

**Figure 9:**
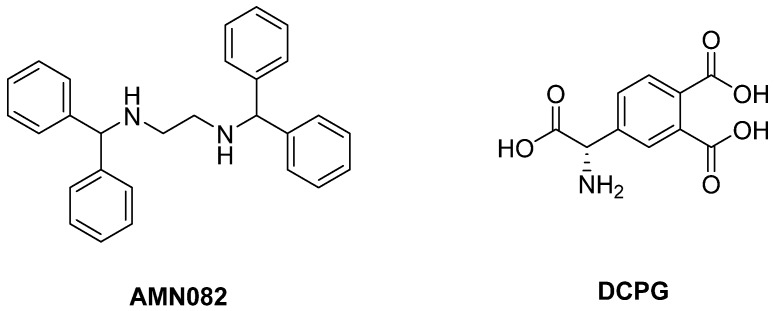
mGlu7R and mGlu8R ligands.

The role played by mGlu7R and mGlu8R in PD and LID need to be elucidated as highly potent and selective ligands have become available only recently and have not been fully pharmacologically characterized yet. Only **AMN082**, a selective mGlu7R allosteric agonist [206], was shown to have some modest antiparkinsonian effects in reserpine-induced akinesia [207] as well as in haloperidol-induced akinesia animal models [208] (Figure 9).

The mGlu8R agonist **DCPG** failed to have antiparkinsonian effect in rodent models of PD [207]. However, other studies reported that this compound elicited a reduction of haloperidol-induced catalepsy and reserpine-induced akinesia but only when Haloperidol or Reserpine are administered for a prolonged period of time [209] in 6-OHDA-lesioned rats. This evidence highlighted the need for further studies to understand the mechanisms underlying the antiparkinsonian effects of DCPG.

## 4. Noradrenergic Receptors

The noradrenergic system plays an important role in the pathophysiology of PD. Noradrenergic neurons in the locus coeruleus [210] undergo degeneration in PD and may even anticipate the death of DA neurons [211,212,213]. They appear to play a protective role by establishing the extent of nigral degeneration induced by both neurotoxic damage and pathological events underlying PD [212,214,215]. Therefore, the indirect activation of adrenergic pathways by blocking presynaptic α_2_ adrenergic autoreceptors should prevent the nigrostriatal DA degeneration and subsequent motor deficits in PD. 

Moreover, being the noradrenergic system implicated in autonomic function, targeting α_2_ or β adrenergic receptors (α_2_-Ars or β-Ars, respectively) appears to have potential to improve symptomatic orthostatic hypertension in PD. 

### 4.1. α_2_-Ars

Stimulation of α_2_-Ars overexpressed in striatal GABAergic neurons activates direct basal ganglia pathway and is involved in the generation of LID, justifying the investigation of α_2_-AR antagonists as antidyskinetic agents [216].

**Figure 10:**
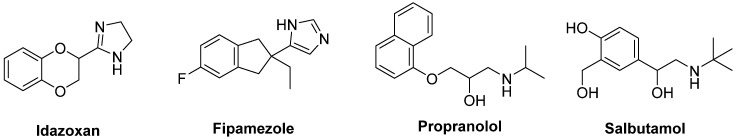
Noradrenergic receptor ligands.

The non-selective α_2_-AR antagonist **Idazoxan** was effective in alleviating the expression of AIMs in 6-OHDA-lesioned rats [217] (Figure 10). In a randomized, placebo-controlled pilot study, Idazoxan improved the severity of LIDs without affecting the antiparkinsonian effect of LD [218], but increasing the frequency of cardiovascular side effects. 

**Fipamezole**, a more recently developed α_2_-AR antagonist, has also been shown to extend both the duration and quality of LD action in MPTP-lesioned NHP [219]. A clinical trial with ten PD patients has demonstrated good tolerability and sound antidyskinetic effect [220]. In a Phase II double-blind, placebo-controlled study in US and Indian PD patients Fipamezole did not show any significant antidyskinetic effect. However, the analysis of US subjects revealed that it reduced LIDs in a dose-dependent manner with an acceptable profile of adverse effects [221]. Other clinical trials with Fipamezole have been performed but the results have not been published yet (NCT01149811, NCT01140841, NCT00040209).

### 4.2. β-ARs

Pharmacological and neuroanatomical evidences support a role for β-ARs as potential therapeutic targets against LID. Indeed, both β_1_- and β_2_-ARs are expressed in the striatum [222] and are integral in PD patients [223].

The β_2_-AR antagonist **Propranolol** has been reported to reduce LID without affecting LD’s efficacy in several experimental and clinical studies (Figure 10). However, it failed to reduce dyskinesia produced by the D_1_ receptor agonist SKF81297 or the D_2_ receptor agonist Quinpirole. Antidyskinetic properties of Propranolol appear to be mediated via attenuation of LD-induced extra-physiological efflux of DA [224]. β blockers might be preferred first-line agents in PD patients who has co-morbid hypertension. Moreover, they are associated with a lower risk of constipation, which is one of the most frequent non-motor symptoms of PD [225]. However, in patients with asthma or chronic obstructive pulmonary disease, β blockers should not be used owing to the risk of bronchospasm [226]. 

Evidences also support the use of β_2_-AR agonists in PD therapy. Indeed, from molecular and immunological studies adrenergic stimulation has been suggested to decrease both α-synuclein deposition and release of neurotoxic molecules. In small clinical trials the β_2_-AR agonist **Salbutamol** in combination with LD improved parkinsonian symptoms in patients with fluctuating PD. Nevertheless, large randomized controlled trials are lacking [227].

## 5. Adenosine Receptors

Adenosine is a neuromodulator that regulates responses to DA and other neurotransmitters in areas of the brain that are responsible for motor function as well as learning and memory [228]. While the monotherapy with adenosine receptor antagonists reaches limited efficacy in the treatment of PD, their use as coadjuvants to LD appears to be a promising strategy.

### 5.1. A_1_ Receptors

**Figure 11:**
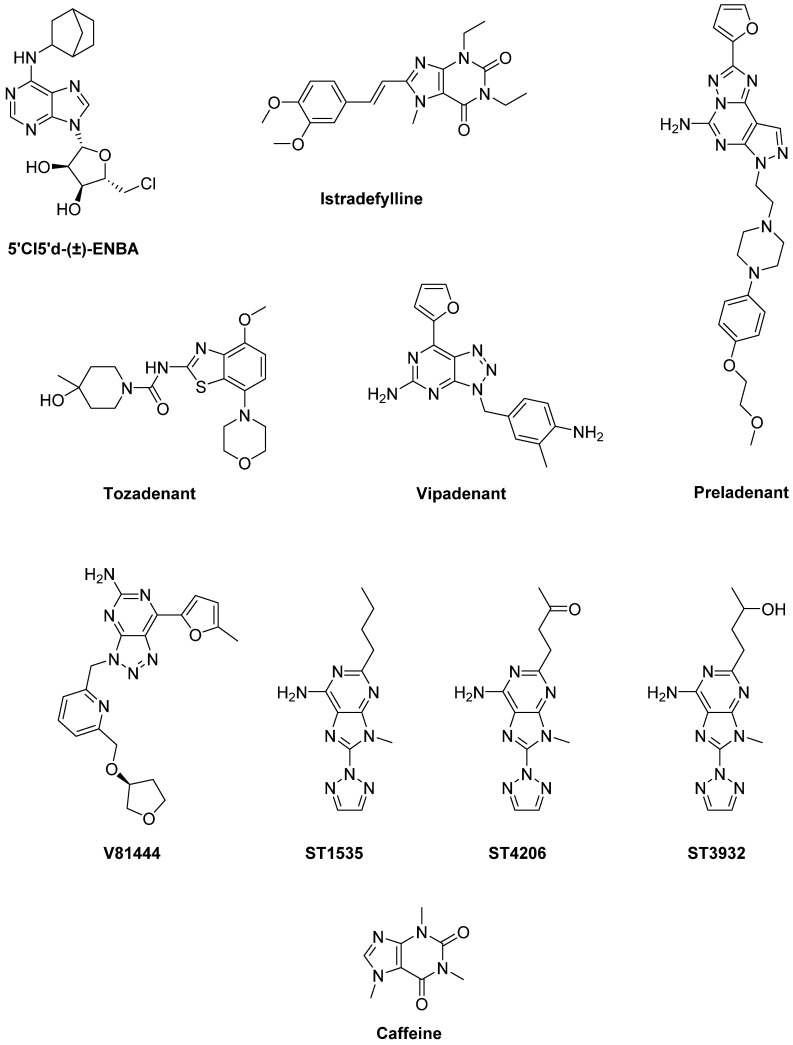
Adenosine receptor ligands.

Adenosine has been reported to antagonize D_1_ receptor-mediated transmission through the stimulation of A_1_ receptors [229,230], which are widely expressed in the substantia nigra pars reticulate [231]. The selective adenosine A_1_ receptor agonist **5′Cl5′d-(±)-ENBA**, administered in combination with LD, reduced the development of AIMs, indicating the potential efficacy of A_1_ agonists for the treatment of LID and hyperkinetic disorders [232] (Figure 11).

### 5.2. A_2A_ Receptors

A_2A_ receptors are highly expressed and co-localized with D_2_ and D_3_ receptors on striatopallidal output neurons in the striatum. Activation of A_2A_ receptors causes the hetero-dimerization with D_2_ receptors and inhibits indirect basal ganglia pathway from striatum to thalamus [233,234]. As demonstrated by preclinical and clinical studies, A_2A_ receptor antagonists are able to improve motor dysfunctions of PD while reducing side effects such as dyskinesia [235].

**Istradefylline** (KW6002), one of the first selective A_2A_ antagonists tested in clinics, received marketing approval in Japan in 2013 for the treatment of PD [236] (Figure 11). In preclinical studies, administration of Istradefylline to 6-OHDA-lesioned rats, previously exposed to LD and exhibiting AIMs with each LD intake, did not elicit AIMs. Moreover, Istradefylline didn’t increase AIMs when administered with LD. However, it didn’t enhance the antiparkinsonian action of LD, assessed by the rotarod performance [237]. In the MPTP-lesioned marmoset, administration of Istradefylline reversed parkinsonism similarly to LD, without eliciting dyskinesia [238] and increasing motor activity [239]. When Istradefylline was administered to MPTP-lesioned marmosets previously treated with LD, it enhanced the antiparkinsonian action of the D_2_ agonist Quinpirole and, at lesser extent, of the D_1_ agonist SKF-80,723 [240]. The combination of Istradefylline and a low dose of LD caused a reduction of LID after chronic treatment, while maintaining the antiparkinsonian effect [241]. In the MPTP-lesioned macaque, Istradefylline monotherapy reduced parkinsonism [242]. After exposure to LD, it reversed parkinsonian disability without eliciting dyskinesia. Finally, when administered with sub-active dose of LD, Istradefylline did not enhance the antiparkinsonian action and dyskinesia, but specifically alleviated bradykinesia [243,244]. Taken together, these data support the clinical use of Istradefylline as co-adjuvant in PD therapy to manage various LD-induced complications. Istradefylline has been found to improve LD-related motor complications in many clinical trials. Some Phase II and III studies showed significant OFF time reduction in PD patients [245,246,247,248]. In contrast, in a large study it failed to demonstrate significant OFF time reduction [249]. Nevertheless, a meta-analysis of all randomized trials concluded that Istradefylline is clinically useful for increasing ON time and reducing OFF time in PD patients with motor fluctuations [250], as supported by a subsequent Phase III trial performed in patients with advanced PD [251]. The findings of an analysis of a post-marketing surveillance study are comparable with previous pre-approval clinical trials in Japan, demonstrating safety and effectiveness of Istradefylline in LD-treated PD patients with the wearing-off phenomenon [252]. Overall, from most of clinical data reported to date, Istradefylline demonstrated to be a well-tolerated and easy to use drug which shows efficacy in advanced PD patients without significantly increasing dyskinesia. This compound might represent a valid adjuvant in LD and other dopaminergic drug therapy to maximize their efficacy and minimize motor fluctuations [253]. Other clinical trials are currently ongoing to confirm the efficacy of Istradefylline in moderate to advanced PD patients (NCT01968031, NCT02610231).

**Preladenant** (SCH-420,814/MK-3814), another selective competitive A_2A_ receptor antagonist, reversed parkinsonian disability without eliciting dyskinesia in rodent and primate models of PD [254,255]. When added to a low dose of LD, it enhanced its antiparkinsonian effect, without increasing LID [254]. In two Phase II trials evaluating Preladenant in combination with LD in PD patients for 12 or 36 weeks, a significant OFF time reduction was shown [256,257]. On the contrary, in other Phase III and Phase II trials, Preladenant failed to elicit the same effect probably owing to inappropriate study design and execution [258,259,260]. 

The A_2A_ antagonist **Tozadenant** (SYN115) could also alleviate motor fluctuation. Indeed, in a Phase IIa study it elicited faster tapping speed before and during LD infusion compared to placebo [261] and, in a Phase IIb trial it was effective in reducing OFF time [262]. A Phase III study, assessing safety and efficacy of Tozadenant to treat end of dose wearing-off in PD patients using LD is currently ongoing (NCT02453386). 

**Vipadenant** (V2006, BIIB014) is also a selective A_2A_ antagonist which has reached clinical trials for the treatment of PD. This compound reduced OFF time duration and extended ON time in PD patients, without troublesome dyskinesia. However, 41% of Vipadenant-treated patients experienced adverse effects [263].

Other A_2A_ antagonists that have progressed to Phase I clinical trials include **V81444**, **PBF-509** (structure not disclosed), **ST1535** and its metabolites **ST4206** and **ST3932**. V81444 is an A_2A_ antagonist currently under development. In a Phase I study it showed rapid absorption when orally administered, a half-life compatible with twice daily dosing, and minimal urinary excretion [264]. Moreover, a Phase Ib/II study is ongoing (NCT01634568). PBF509 potentiated the activity of LD in reversing parkinsonian motor impairments and inhibited LID in 6-OHDA-lesioned rats [265]. In a Phase I clinical trial (NCT01691924) it showed safety, tolerability and feasibility. ST1535 enhanced LD-induced rotational behavior in 6-OHDA-lesioned rats [266,267] and potentiated the antiparkinsonian action of a sub-active dose of LD in MPTP-lesioned marmosets [268]. A Phase I clinical trial demonstrated that ST1535 was well tolerated [269]. Its metabolites ST4206 and ST3932 showed a similar pharmacological activity and may be considered potentially therapeutic alternatives to ST1535.

### 5.3. A_1_/A_2A_ Receptors

The non-specific adenosine receptor antagonist **Caffeine** has shown antiparkinsonian and neuroprotective effects in animal models of PD [270,271,272] (Figure 11). A clinical trial demonstrated that Caffeine could reduce the probability of developing dyskinesia [273]. However, in another randomized trial no significant changes in motor features were observed [274]. A Phase III trial to evaluate the efficacy of Caffeine in PD is currently ongoing (NCT01738178).

## 6. Histamine Receptors

Histamine H_2_ and H_3_ receptors are highly expressed in basal ganglia and might be involved in motor activity, thus representing another potential target for the treatment of LID in PD patients [275].

### 6.1. H_2_ Receptors

H_2_ receptors are mainly distributed in basal ganglia, particularly in the striatum. In mouse models the activation of cholinergic interneurons in LID has been demonstrated to be inhibited by blocking H_2_ histaminergic transmission, providing a strong rationale to reduce LID in PD patients by targeting such receptors [276].

**Figure 12:**
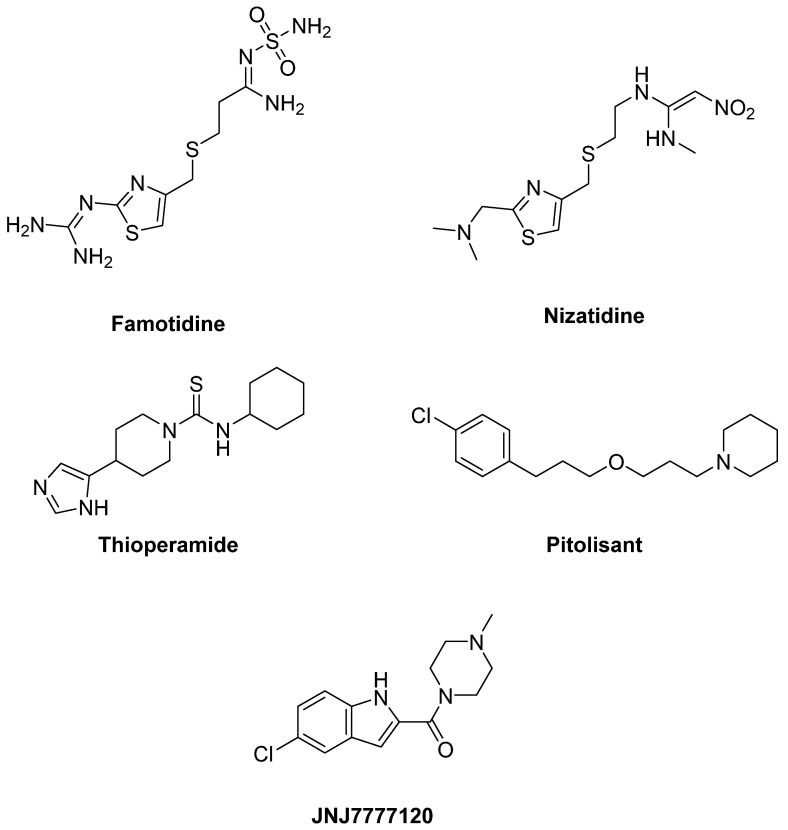
Histamine receptor ligands.

The selective H_2_ antagonist **Famotidine** enhanced the antiparkinsonian effects of LD and reduced LID in two mouse models [276] and a primate model of PD [277] (Figure 12). However, a Phase II trial evaluating Famotidine failed to demonstrate efficacy in reducing dyskinesia severity, although this trial used relatively low doses [278]. 

In PD patient, **Nizatidine**, another selective H_2_ antagonist, demonstrated to be efficacious in ameliorating gastroparesis and slow transit constipation [279,280].

### 6.2. H_3_ Receptors

The observation that the H_3_ antagonist **Thioperamide** potentiated DA agonist-induced locomotor activation suggested a potential benefit of H_3_ antagonists on motor control in PD patients [281]. Thioperamide was also demonstrated to counteract memory and sleep impairment in a 6-OHDA-lesioned mouse model of PD [282] (Figure 12).

**Pitolisant**, the only H_3_ inverse agonist approved for the treatment of narcolepsy with and without catalepsy, is in Phase III clinical trials for the treatment of excessive daytime sleepiness in PD patients (NCT01036139, NCT01066442, NCT00642928).

### 6.3. H_4_ Receptors

The activity of microglia, which is regulated by H_4_ receptors, seems to play a key role in the pathogenesis of PD. Accordingly mRNA expression of H_4_ receptors proved to be increased in the basal ganglia of PD patients [283]. In rotenone-induced PD rat model the specific H_4_ antagonist **JNJ7777120** blocked the microglial activation, reduced apomorphine-induced rotational behavior, prevented decreases in striatal DA levels, providing the first evidence of the efficacy of an H_4_ antagonist in PD [284] (Figure 12).

## 7. Cholinergic Receptors

It is well known that DA action is contrasted by acetylcholine (ACh) in striatum and unbalanced signaling between these neurotransmitter systems could alter basal ganglia activity and motor function, as it occurs in PD and LID. Numerous studies show that in PD nigrostriatal damage with severe DA depletion causes abnormal increase in cholinergic interneurons activity that, via strategically positioned nicotinic and muscarinic ACh receptors, promote striatal signaling to attenuate normal movements. Recently, new technology and pharmacological agents have facilitated understanding the role of ACh transmission in PD and LID, thus offering new therapeutic strategies in movement disorders [285,286,287].

### 7.1. Muscarinic Receptors

**Figure 13:**
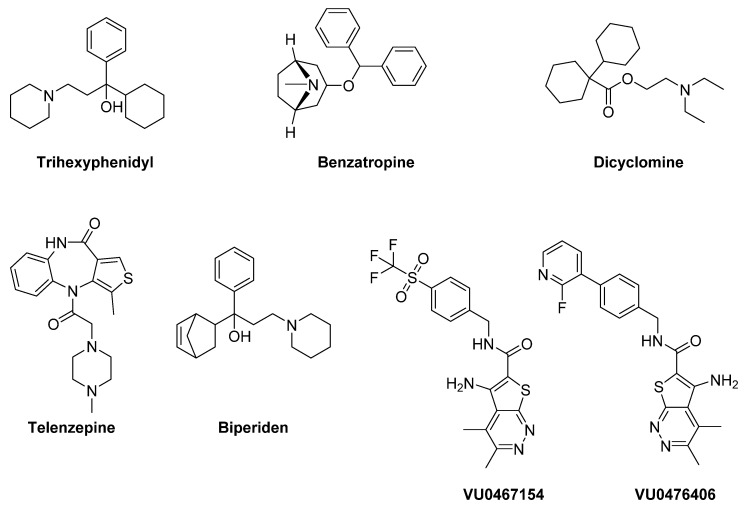
Muscarinic receptor ligands.

Muscarinic cholinergic antagonists have been considered in the treatment of PD for decades [288]. They are effective in preventing acute dyskinesias, especially in young patients. However, poor subtype selectivity and the occurrence of severe side-effects (confusion, hallucination, dry mouth, memory disturbance, urinary retention) have limited their use [289,290]. Trihexyphenidyl (benzhexol) has been considered one of the most representative compounds within this class. It inhibits the excitability of cholinergic neurons by blocking striatal M_1_ receptors. Although it was taken into account for the treatment of PD since 1949, only quite recently it was approved by the FDA for the treatment of parkinsonian tremor, dyskinetic movements and spastic contractions [291] (Figure 13). When the above-mentioned forms of parkinsonism are treated with LD, Trihexyphenidyl is often used as an adjuvant therapy [291]. 

The M_1_ receptor antagonist **Benzatropine** also demonstrated to be efficacious for PD tremors [292]. Moreover, the non-subtype selective muscarinic antagonist **Dicyclomine** proved to enhance LD’s antiparkinsonian effects and to significantly attenuate LID in Pitx3-deficient aphakia mice [293].

M_1_ muscarinic receptors may also play a role in the modulation of PD non-motor deficits. Indeed, the preferential M_1_ antagonist **Telenzepine** demonstrated to improve anxiety-like behavior and social memory recognition in 6-OHDA-lesioned mice, suggesting that dysfunction of the striatal cholinergic system affects emotional and cognitive deficits in mice with reduced DA levels [294]. 

**Biperiden** is another muscarinic receptor antagonist with high affinity for the M_1_ subtype used in the treatment of PD and neuroleptic-induced extrapyramidal motor side effects. Recently, it has also been demonstrated to behave as a weak inhibitor of acetylcholinesterase [295].

Recent studies report that the M_4_ PAMs **VU0467154** and **VU0476406** significantly attenuated dyskinetic behaviors in mouse and primate models of LID in PD. These results suggest that activation of M_4_ muscarinic receptors, facilitating long-term depotentiation in D_1_ medium spiny projection neurons, might represent a novel pharmacological strategy to alleviate LID in PD patients [296].

### 7.2. Nicotinic Agonists

Activation of nicotinic receptors expressed on dopaminergic neurons indirectly affects DA release [297,298]. Moreover, it can also indirectly modulate GABA, serotonin and glutamate release, since nicotinic receptors are also localized on GABAergic, serotoninergic and glutamatergic interneurons [299,300]. Preclinical evidence demonstrated that nicotinic receptor ligands reduced LID by up to 60% in different PD animal models [285,287]. However, clinical studies on the involvement of the nicotinic system in LIDs are only emerging [301]. Interestingly, both nicotinic receptor agonists and antagonists similarly demonstrated to reduce LIDs in PD animal models. This can be due to the fact that prolonged exposure to agonist can lead to nicotinic receptor desensitization, ultimately reducing neurotransmitter release [297,302,303,304,305]. Therefore, nicotinic agonists and antagonists induce a similar functional blockade [306]. 

**Figure 14:**
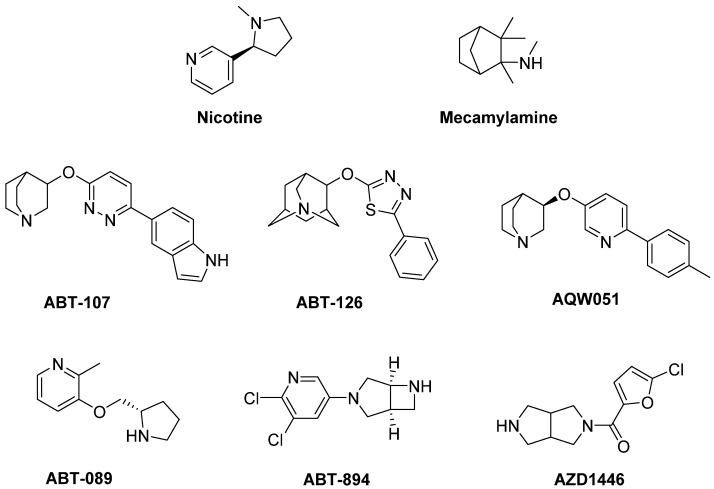
Nicotinic receptor ligands.

In addition to providing neuroprotection, **Nicotine** also demonstrated to protect against LID in different models of PD [306,307,308,309,310,311,312,313,314,315,316,317] (Figure 14). 

Interestingly, similar effects were observed with the non-selective nicotinic receptor antagonist **Mecamylamine** [306,310]. In clinical trials Nicotine showed antidyskinetic effect in PD patients after oral treatment [318,319]. Results of a small Phase II trial have never been published (NCT00957918).

Preclinical studies have shown that the nicotinic receptor subtypes α7 and β2* (the asterisk indicates the possible presence of other subunits in the receptor complex) are mainly implicated in mediating both neuroprotective and antidyskinetic effects, suggesting that nicotinic subtype selective drugs may be beneficial therapeutic agents for LID management [300,308,320]. The α7 nicotinic receptor agonists **ABT-107** and **ABT-126** significantly reduced LID in PD monkeys without developing tolerance or worsening parkinsonism. ABT-126 was also effective in monkeys with both severe and moderate nigrostriatal damages, suggesting its ability to reduce dyskinesias in early- and later-stage PD [321,322].

Analogously, the selective α7 nicotinic receptor partial agonist **AQW051**, studied in MPTP-lesioned monkeys, reduced LID and extended LD antiparkinsonian response [323]. However, it failed to reduce dyskinesia or parkinsonian severity in idiopathic PD patients [324]. Several β2* nicotinic receptor subtype agonists, including **ABT-089**, **ABT-894,** and **AZD1446**, also demonstrated to significantly reduce LID in most dyskinetic animals without worsening parkinsonism and developing tolerance [325,326]. The extent of LID reduction didn’t increase by co-administration of α7 and β2* nicotinic receptor subtype agonists with respect to the drugs administered alone, suggesting that they act through a common mechanism of action [321]. Overall, the use of compounds selectively targeting β2* or α7 subtype appears to be a good therapeutic approach to alleviate LID. Thus, both classes of drugs may be promising antidyskinetic agents to be tested in clinical trials. 

Since attenuated dopaminergic neurodegeneration and motor dysfunction have been observed in hemiparkinsonian α5-KO mice, nicotinic receptors containing the α5 subunit represent potential novel targets in the treatment of PD [327].

## 8. GABA Receptors

Considering that alterations in GABAergic neurotransmission may contribute to some of the axial symptoms of PD [328], GABA modulation has been proposed as a new strategy for PD treatment [329].

**Figure 15:**
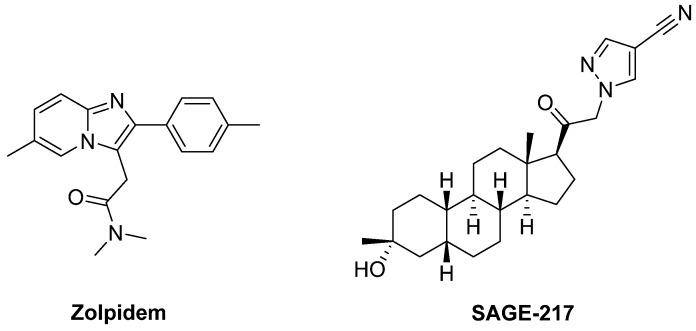
GABA receptor ligands.

The GABA_A_ receptor agonist **Zolpidem**, a PAM with selective affinity for receptors expressing the α1 subunit, improved motor impairments in unilateral 6-OHDA-lesioned rats, suggesting that targeting Zolpidem-sensitive GABA_A_ receptors may be a novel approach to treat motor symptoms in PD [330] (Figure 15). 

**SAGE-217**, another GABA_A_ receptor PAM, is an orally bioavailable steroidal derivative which has reached a Phase II trial for the treatment of PD as monotherapy or in combination with LD. The results are not available yet (NCT03000569).

## 9. Neurokinin Receptors

**Figure 16:**
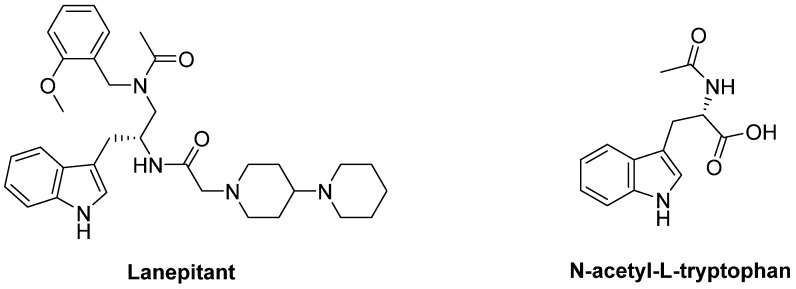
Neurokinin receptor ligands.

The abnormal stimulation of DA receptors, associated with LID and AIMs, induces up-regulation of FosB expression in dynorphin containing striatal cells where substance P (SP) is co-localized. LD treatment proved to increase SP in the substantia nigra. SP receptor antagonists has been suggested to reduce LID by blocking neurokinin 1 (NK1) receptors. Indeed, in 6-OHDA-lesioned rats the NK1 antagonists **Lanepitant** (LY303870) and **N-acetyl-L-tryptophan** demonstrated to ameliorate LID without affecting the therapeutic effect of LD and conserving motor function [331,332] (Figure 16).

## 10. Opioid Receptors

Opioid receptors, especially δ receptor subtype, and the endogenous opioid peptides enkephalin and dynorphin are expressed in basal ganglia and cortex, where the opioid system modulates the activity of spiny projection neurons in motor disorders such as PD [333,334]. The level of opioid peptides demonstrated to be increased in the striatum, thalamus and anterior cingulate cortex [225] in PD animal models and PD patients exhibiting dyskinesia. Therefore, selective agonists and antagonists of opioid receptors have been used to contrast akinesia and LID in PD [335]. Moreover, due to the well known involvement of opioid in pain, several studies have investigated their potential for the treatment of pain in PD [288]. 

**Figure 17:**
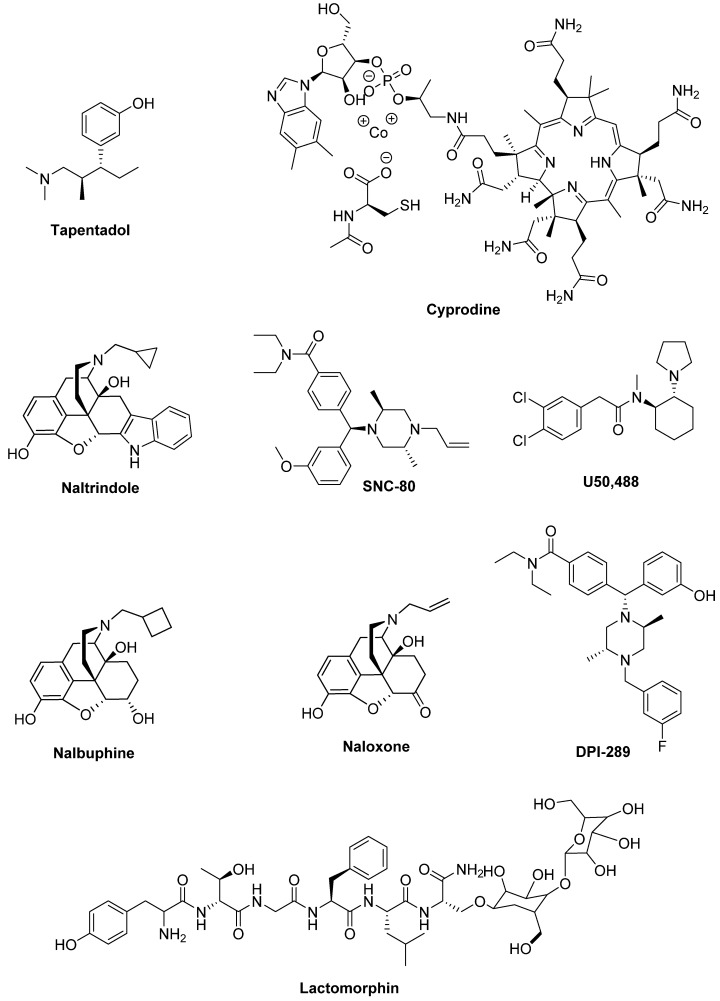
Opioid receptor ligands.

**Tapentadol**, a µ opioid receptor agonist with a serotonin/noradrenaline reuptake inhibitor activity, efficaciously reduced pain and was well tolerated in PD patients [336] (Figure 17).

The µ opioid receptor antagonists might also be involved in PD therapy. Indeed, in MPTP-lesioned NHP **Cyprodine** and **ADL5510** (structure not available) reduced LID without affecting the antiparkinsonian effects of LD [337,338].

A similar effect was induced by the selective δ antagonist **Naltrindole**, which demonstrated to alleviate LID in MPTP-lesioned marmoset and 6-OHDA-lesioned rats [337,339], while the δ agonist **SNC-80** increased locomotor activity in PD animal models [340,341,342]. On the contrary, the selective κ receptor agonist **U50,488** reduced LID in rat and monkey models of PD, although it contrasted the anti-parkinsonian effects of LD [343].

Analogously, the κ agonist and µ antagonist **Nalbuphine** alleviated LID in an NHP model of PD and decreased the levels of specific dyskinetic molecular markers [344]. 

Contrasting results were obtained with the non-selective opioid antagonist **Naloxone**, which reduced LID in 6-OHDA-lesioned rats [114,345], while the same effect was not observed in NHP and PD patients [346,347].

Particularly interesting are the results obtained with **DPI-289** a δ Agonist/µ Antagonist (DAMA), which provided anti-parkinsonian action in rodent and NHP models of PD both alone or in combination with LD, without increasing dyskinesia, thus representing an LD-sparing strategy for clinical development [348].

The glycosylated derivative of the opioid peptide Leu-enkephalin **Lactomorphin** (MMP-2200) [H_2_N-Tyr-D-Thr-Gly-Phe-Leu-Ser-(O-β-d-lactose)-CONH_2_] behaved as a mixed δ/μ opioid receptor agonist [349]. This compound showed a modest antiparkinsonian activity, but reduced dyskinesia induced by D_2_-like receptor agonists [165]. A study evaluating Lactomorphin in combination with the NMDA receptor antagonist MK-801 is reported in the section “glutamate receptors”. 

## 11. Sigma-1 (σ_1_) Receptors

σ_1_ Receptor is a type of non-opioid receptor [350] that is down-regulated in the brains of early stage PD patients [351,352]. Recently, the pharmacological stimulation of such a receptor has shown improvement of LID and neurorestorative and protective properties in experimental PD models [352,353]. 

**Figure 18:**
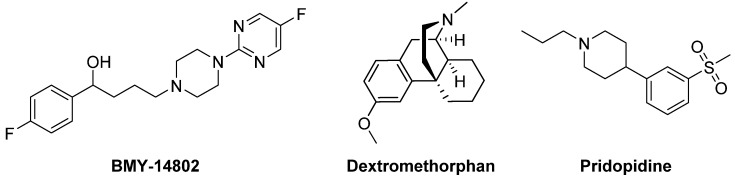
σ_1_ receptor ligands.

σ_1_ Receptor ligands, such as the antagonist **BMY-14802** [354], have been reported to be potentially useful for the treatment of LID [355] (Figure 18). 

The σ_1_ receptor agonist **Dextromethorphan** caused a reduction of dyskinesia by about 30–40%, without affecting the beneficial effect of LD [356]. This compound, that also behaves as a non-competitive NMDA receptor antagonist, as well as a serotonin and norepinephrine reuptake inhibitor is rapidly metabolized by hepatic cytochrome P450 CYP2D6. A Phase IIa clinical trial (NCT01767129) provided preliminary evidence of the efficacy, safety, and tolerability of Dextromethorphan in combination with the potent CYP2D6 inhibitor Quinidine for the treatment of LID in PD patients [357]. However, further studies with a longer treatment duration are needed to validate these early findings.

**Pridopidine**, a small molecule under development for the treatment of Huntington’s disease [358], produced a significant decrease in LID maintaining the antiparkinsonian benefit of LD in MPTP-lesioned macaques. Although such an effect was associated with full σ_1_ occupancy, such a mechanism alone is unlikely responsible for the antidyskinetic efficacy of Pridopidine which may be associated with the involvement of non-σ receptors [358].

## 12. Conclusions

**Figure 19:**
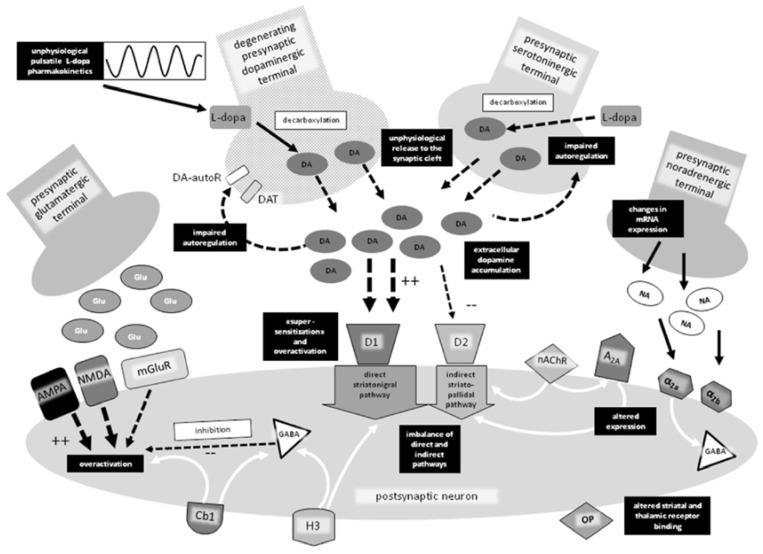
Pathophysiology of levodopa-induced dyskinesia (Reprinted with permission from Springer Nature: Springer *CNS Drugs* Pharmacological Strategies for the Management of Levodopa-Induced Dyskinesia in Patients with Parkinson’s Disease, Schaeffer, E.; Pilotto, A.; Berg, D., **2014**, doi: 10.1007/s40263-014-0205-z. [360]). 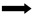 Physiological activation, 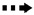 pathological alterations in LD-induced dyskinesia (LID), 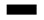 pathological alterations in LID, 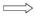 modulation, ++ increased activation, -- decreased activation. *A_2A_* adenosine receptor, *α_2a_* and *α_2ab_* noradrenergic receptors, *AMPA* α-amino-3-hydroxy-5-methyl-4-isoxazolepropionic acid, *Cb_1_* cannabinoid receptor, *D_1_* and *D_2_* dopaminergic receptors, *DA* dopamine, *DA-autoR* dopamine autoreceptor, *DAT* dopamine transporter, *GABA* γ-aminobutyric acid, *Glu* glutamate, *H_3_* histamine receptor, *LID* levodopa-induced dyskinesia, *mGluR* metabotropic glutamate receptor, *NA* noradrenaline, *nAchR* nicotinic acetylcholine receptors, *NMDA* N-methyl-D-aspartate, *OP* opioid receptor.

Two hundred years ago James Parkinson described in his work “An essay on the shaking palsy” the characteristic of a CNS chronic degenerative disease lately named with his name (PD) [359]. Despite great progresses over the last 200 years, the therapeutic treatment of this disease, which has become the second most diffused neurodegenerative pathology over the world, still remains an unfulfilled dream and a challenge that scientists have to face. The currently available therapies have demonstrated limited efficacy for the following reasons:

- the causes of such a pathology are mostly unknown;
- the dopaminergic system and other receptors, as well as several enzymatic targets not discussed in this review, mutually affect each other and are deeply altered over the course of the disease (Figure 19);
- the same drug used in PD therapy or for the treatment of co-morbidities may aggravate the progression of different disease symptoms;
- the symptoms are individual and fluctuating during the day;
- frequently divergent results come from the experimental models used in the evaluation of drug candidates.

However, the severity of the disease and its increasing diffusion due to the rising of the aged population prompt to the research of new therapeutic tools both administered alone and/or as LD adjuvant. From this point of view, interesting perspectives are given by the discovery of new ligands targeting different receptor systems, which are discussed in this review.

Moreover, based on the evolution of the traditional concept “one molecule-one target” to the newer “one molecule-one disease” that represents a trend of the modern medicinal chemistry, another helpful “stick of the LD old age” may be represented by multitarget ligands, synergistically able to restore dysfunctions of different system.

Considering the numerous possibilities existing in the field of target-based drug discovery, efficacious therapeutic tools might be hopefully available to PD patients in the future.

## Appendix A

PubChem CIDs (or Reaxys IDs) of the compounds reported in the review

**Compound**	**PubChem CID (Reaxys ID)**
5’Cl5’d-(±)-ENBA	15599147
8-OH-DPAT	1220
ABT-089	178052
ABT-107	11151363
ABT-126	24987875
ABT-894	10131048
ADX88178	46836872
Amantadine	2130
AMN082	11698390
ANR 94	11805896
Apomorphine	6005
AQW051	50914822
Aripiprazole	60795
AZD1446	24795080
Befiradol	9865384
Benzatropine	1201549
Biperiden	2381
BMY-14802	108046
Bromocriptine	31101
Buspirone	2477
Cabergoline	54746
Caffeine	2519
CJ-1639	53475319
Clozapine	135398737
CP94253	4029677
Cyprodine	24758534
D-512	(26962985)
D-636	(33944059)
D-653	(33944076)
D-656	(33944078)
DCPG	16062593
DDMPA-8	(33958274)
DETQ	117720272
Dextromethorphan	5360696
Dicyclomine	3042
Dihydroergocriptine	114948
Dipraglurant	44557636
Dizocilpine	180081
DPI-289	(12841869)
Eltoprazine	65853
F13714	(8361393)
F15599	11741361
Famotidine	5702160
Fenobam	135659063
Fipamezole	213041
Foliglurax	135565465
Idazoxan	54459
Ifenprodil	3689
Istradefylline	5311037
JNJ7777120	4908365
Ketamine	3821
L-745,870	5311200
Lactomorphin	Not Available
Lanepitant	3086681
Lisuride	28864
LSP1-2111	46898088
Lu AF21934	66553157
LY379268	10197984
Mavoglurant	9926832
Mecamylamine	4032
Memantine	4054
Mianserin	4184
Mirtazapine	4205
Mosapride	119584
MPEP	3025961
MSX-3	10256041
MTEP	9794218
N-acetyl-L-tryptophan	700653
Nalbuphine	5311304
Naloxone	5284596
Naltrindole	5497186
Nicotine	89594
Nizatidine	3033637
Ondansetron	4595
Perampanel	9924495
Pergolide	47811
Pimavanserin	10071196
Piribedil	4850
Pitolisant	9948102
Pramipexole	119570
Preladenant	10117987
Pridopidine	9795739
Propranolol	4946
Prucalopride	3052762
Pruvanserin	6433122
Quetiapine	5002
Radiprodil	10200813
Ropinirole	5095
Rotigotine	59227
SAGE-217	86294073
Salbutamol	2083
Sarizotan	6918388
SK609	6486733
SLV-308	6918524
SNC-80	123924
ST1535	9860294
ST3932	(20692973)
ST4206	46912314
Talampanel	164509
Tandospirone	91273
Tapentadol	9838022
Tavapadon	86764100
Telenzepine	5387
Tezampanel	127894
Thioperamide	3035905
Topiramate	5284627
Tozadenant	11618368
Traxoprodil	219101
Trihexyphenidyl	5572
U50,488	3036289
V81444	44537963
Vipadenant	21874557
VU0364770	836002
VU0467154	73774630
VU0476406	(23873237)
VU6004461	(29581513)
Zolpidem	5732
